# A Systematic Review of the Preventive and Therapeutic Effects of Naringin Against Human Malignancies

**DOI:** 10.3389/fphar.2021.639840

**Published:** 2021-03-29

**Authors:** Maryam Ghanbari-Movahed, Gloria Jackson, Mohammad Hosein Farzaei, Anupam Bishayee

**Affiliations:** ^1^ Medical Technology Research Center, Health Technology Institute, Kermanshah University of Medical Sciences, Kermanshah, Iran; ^2^ Department of Biology, Faculty of Science, University of Guilan, Rasht, Iran; ^3^ Lake Erie College of Osteopathic Medicine, Bradenton, FL, United States

**Keywords:** citrus fruits, naringin, cancer, therapy, molecular mechanisms, prevention

## Abstract

**Background:** Natural product-based cancer preventive and therapeutic entities, such as flavonoids and their derivatives, are shown to have a noticeable capability to suppress tumor formation and cancer cell growth. Naringin, a natural flavanone glycoside present in various plant species, has been indicated to modulate different signaling pathways and interact with numerous cell signaling molecules, which allows for an extensive variety of pharmacological actions, such as amelioration of inflammation, oxidative stress, metabolic syndromes, bone disorders, and cancer. The purpose of this systematic review is to present a critical and comprehensive assessment of the antitumor ability of naringin and associated molecular targets in various cancers.

**Methods:** Studies were identified through systematic searches of Science Direct, PubMed, and Scopus as well as eligibility checks according to predefined selection criteria.

**Results:** Eighty-seven studies were included in this systematic review. There was strong evidence for the association between treatment with naringin alone, or combined with other drugs and antitumor activity. Additionally, studies showed that naringin-metal complexes have greater anticancer effects compared to free naringin. It has been demonstrated that naringin employs multitargeted mechanisms to hamper cancer initiation, promotion, and progression through modulation of several dysregulated signaling cascades implicated in cell proliferation, autophagy, apoptosis, inflammation, angiogenesis, metastasis, and invasion.

**Conclusion:** The results of our work show that naringin is a promising candidate for cancer prevention and treatment, and might offer substantial support for the clinical application of this phytocompound in the future. Nevertheless, further preclinical and clinical studies as well as drug delivery approaches are needed for designing novel formulations of naringin to realize the full potential of this flavonoid in cancer prevention and intervention.

## Introduction

Cancer is a set of complex processes, including unlimited cell proliferation, death of impaired cells, and spatial-temporal changes in cell physiology, that may result in the formation of malignant tumors with the potential for metastasis (Seyfried and Shelton, 2010). There are many different approaches for the treatment of cancer, but some may be ineffective due to increased resistance to classical anticancer drugs as well as adverse side effects (Abotaleb et al., 2019).

Scientific reports and traditional knowledge demonstrate that a high intake of fruits and vegetables is constantly associated with a decreased risk of some type of human cancers, such as lung, colon, prostate, and breast cancer (Neuhouser, 2004). Fruit-based cancer preventive and therapeutic entities, such as flavonoid and their derivatives, have shown a noticeable capability to suppress tumor formation and cancer cell growth (Ramesh and Alshatwi, 2013). Flavonoids are a big class of natural polyphenols, existing in a broad variety of vegetables and fruits commonly consumed by humans. These phytochemicals are divided into different subclasses, including flavonols, flavan-3-ols, isoflavones, flavanones, anthocyanidins, and flavones (Romagnolo and Selmin, 2012). In the context of carcinogenesis, flavonoids intervene with multiple signal transduction cascades and increase apoptosis as well as inhibit metastasis, angiogenesis, and proliferation (Ravishankar et al., 2013).

Naringin, a flavanone glycoside derived from the flavanone naringenin, is present in many plant species, especially citrus fruits (Zhang et al., 2014). It has been indicated to interact with a wide range of signaling molecules and modulate various signaling pathways and thus has multiple pharmacological impacts, such as antioxidant, anti-inflammatory, antiapoptotic, antitumor, and antiviral properties as well as effects on metabolic syndrome, bone regeneration, neurodegenerative disorders, cardiovascular disease, and genetic damage (Bharti et al., 2014; Chen et al., 2016; Joshi et al., 2018; Rivoira et al., 2020). Notably, previous studies have demonstrated that high dietary intake of naringin reduced the risk of certain cancers, such as lung cancer (Le Marchand et al., 2000). Although a few reports exist on the overview of naringin in cancer, these publications are narrative reviews or reviews of the pharmacological activities of naringin without particular emphasis on its antitumor effects and none of them have evaluated naringin individually in the prevention and treatment of cancer (Meiyanto et al., 2012; Rivoira et al., 2020; Memariani et al., 2020). Hence, a critical and comprehensive systematic review on the anticancer ability of naringin and associated molecular targets within different cancers has not been conducted in the past. Accordingly, the objective of this article is to present a critical and up-to-date systematic evaluation of the preventive and therapeutic impacts of naringin and associated cellular and molecular mechanisms of action.

## Natural Products and Malignancies

It is known that throughout history, natural products have played an important role in health promotion and disease prevention. Natural products represent a valuable resource in the development and discovery of new drugs, particularly those used for cancer treatment (Newman and Cragg, 2012; Cragg and Pezzuto, 2016; Newman and Cragg, 2020). A large number of the significant advances in cancer treatment are directly associated with the development of natural product-based drugs and the use of these agents to suppress, reverse, or retard the process of carcinogenesis (Cragg and Pezzuto, 2016). Many natural products from herbs, vegetables, plant extracts, and fruits exert chemoprotective properties against carcinogenesis (Amin et al., 2009; Gullett et al., 2010; Lee et al., 2011; Bishayee and Sethi, 2016). Plant secondary metabolites, also known as phytochemicals, belong to four major classes, such as terpenoids, phenolics, alkaloids, and sulfur-containing compounds. A large number of these phytocompounds are strong antioxidants as well as anti-inflammatory agents with reactive groups that confer protective activities. The vast majority of the non-nutrient antioxidants present in various plants are phenolic compounds, including catechins in tea, isoflavones in soybeans, phenolic esters in coffee, quercetin in onions, phenolic acid in red wine, and rosmarinic acid in rosemary (Sheikh et al., 2021). Flavonoids, a subclass of polyphenols, have also been demonstrated to block the cell cycle progression, protect cells from damage due to external factors, suppress mutations, inhibit prostaglandin synthesis, and inhibit carcinogenesis in animal models (Abdulla and Gruber, 2000). Several animal studies have shown a protective effect for isoflavonoids against mammary cancers (Steiner et al., 2008; Basu and Maier, 2018; Ávila-Gálvez et al., 2020). A high isoflavone diet has also been indicated to suppress tumorigenesis in various animal models for prostate cancer (Persky et al., 1994). Multiple lines of experimental evidences suggest that treatment with naringenin or novel naringenin formulations could inhibit various malignancies, such as melanoma, breast, and cervical cancer (Krishnakumar et al., 2011; Rajamani et al., 2018; Choi et al., 2020). Tea is an essential source of flavonols and flavanols. Many experimental studies show an anticancer effect for tea polyphenols (Yang et al., 2001). It has also been indicated that administration of genistein early in life increases the differentiation and early maturation of the rat mammary gland (Persky et al., 1994), conferring protection against breast cancer. Although synthetic cancer drugs cause non-specific cell killing, natural products, including dietary phytochemicals, offers therapeutic and protective activities with low cytotoxicity (Reddy et al., 2003).

## Naringin: Sources, Chemistry, and Pharmacology

**FIGURE 1:**
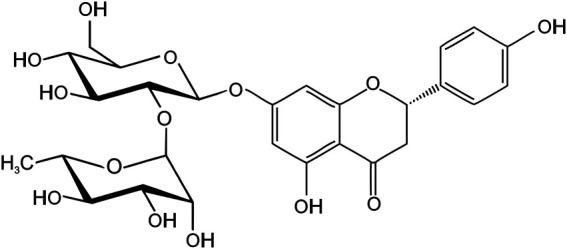
The chemical structure of naringin.

Naringin, chemically known as 4′,5,7-trihydroxyflavanone-7-rhamnoglucoside (C_27_H_32_O_14_, molecular weight: 580.5, Figure 1), is a flavone glycoside that is present in many plant species, particularly citrus fruits, with remarkable pharmacological and biological activities. It is one of the main active components of various Chinese herbal medicines, such as *Citrus medica L*. (CM) and *Citrus aurantium L*. (CA) (Table 1) (Alam et al., 2014; Zhang et al., 2014). The chemical structure of naringin was first annotated in 1928 by Inubuse and Asahina (EFSA, 2011). In one study, naringin was isolated from *C. aurantium* crude peel extract after HPLC separation and its structure was confirmed by electrospray ionization mass spectrometry. The predicted mass for naringin was 580 Da (Zhang et al., 2018a). Naringin is derived from naringenin and is responsible for the bitterness of citrus fruits and their products (Konno et al., 1982). It can be hydrolyzed by rhamnosidase activity of naringinase into prunin and rhamnose, which can be further hydrolyzed by the b-D-glucosidase component of naringinase, into naringenin and glucose (Real et al., 2007).

**TABLE 1:** Various natural sources of naringin.

Source plant	Naringin content (μg/ml)	References
*Citrus × aurantium* L. [Rutaceae]	19.7	Kawaii et al. (1999)
Citrus × limon (L.) Osbeck [Rutaceae]	22.3	Kawaii et al. (1999)
Citrus deliciosa Ten. [Rutaceae]	8.0	Dhuique-Mayer et al. (2005)
Citrus medica L. [Rutaceae]	18.6	Menichini et al. (2016)
Citrus × aurantium L. [Rutaceae]	230.0	Kawaii et al. (1999)
Citrus × aurantium L. [Rutaceae]	3383.6	de Lourdes Mata Bilbao et al. (2007)
Citrus × aurantium L. [Rutaceae]	21.3	Ooghe et al. (1994)

Naringin has been shown to modulate various enzyme and protein expressions, thus exerting potential therapeutic activities. Naringin has been demonstrated to significantly affect cell proliferation and osteogenic differentiation (Dai et al., 2009). Naringin has also been indicated to be effective in decreasing the expression of numerous signaling factors involved in the inflammatory response, e.g., interleukin-8 (IL-8), tumor necrosis factor-α (TNF-α), interleukin-6 (IL-6), inducible nitric oxide synthase (iNOS), and nuclear factor erythroid 2-related factor 2 (Nrf2) (Habauzit et al., 2011). It has also been reported to decrease metabolic syndrome through downregulation of the expression of key gluconeogenic enzymes and upregulation of AMP-activated protein kinase. Additionally, it decreases the activity of 3-hydroxy-3-methylglutaryl coenzyme A reductase and enhances the production of nitric oxide metabolites. Naringin also shows antigenotoxic actions and decreases DNA damage by controlling the generation of free radicals and the expression of oxidative mediators (Chen et al., 2016). It has beneficial effects on many central nervous system diseases, including epilepsy, Parkinson’s disease, and Alzheimer’s disease (Jäger and Saaby, 2011), and has been demonstrated to have dose-dependent radical scavenging activity and decreased oxidative stress (Rajadurai and Prince, 2007). Overall, naringin can be regarded as a promising natural compound that elicits various health benefits.

## Methodology for Literature Search on Naringin and Malignancies

### Search Strategy

The current systematic review was conducted following the Preferred Reporting Items for Systematic Reviews and Meta-Analysis (PRISMA) guidelines (Moher et al., 2009), employing several electronic databases (Science Direct, PubMed, and Scopus) and using the following keywords: “naringin” AND (“neoplasm” OR “cancer” OR “tumor” OR “carcinoma” OR “malignancy”). The information about populations, interventions, comparators, outcomes, and study designs (PICOS) criteria are presented in Table 2.

**TABLE 2:** Description of population, intervention, comparator, outcome and study design (PICOS).

Population	•Normal and cancer cell lines
	•Healthy and tumor bearing animals
Intervention	•Naringin
Comparison	•∆-changes between treatments (naringin/control/anticancer drug)
Outcome	•Effect of naringin on cancer cell growth inhibition and/or reduction of tumor size and volume
Study design	•*In vitro* studies
	•*In vivo* studies

### Inclusion Criteria

Experimental studies (*in vivo* and *in vitro*) up to September 2020 in the English language which assessed the anticancer effect of naringin (in any cancer cell line and/or animal model) were included.

### Exclusion Criteria

We applied the following exclusion criteria: 1) conference abstracts, books, book chapters, and unpublished results; 2) non-English papers; 3) reviews, systematic reviews, meta-analysis, and letters; 4) primary research papers that do not involve tumor cell lines or animal tumor models.

### Data Extraction

Among the initial 2,998 reports that were collected through electronic search, 137 were omitted due to duplicated results, 985 were ruled out because of the article type, 613 review articles were omitted, and 952 were deemed irrelevant based on abstract and/or title information. Besides, 18 were excluded because they were not in English language. Out of 293 retrieved reports, 97 were excluded as they evaluated the whole plant, 75 were ruled out as they examined other biological impacts of naringin rather than anticancer effects, and 34 were excluded because they concentrated on other compounds, not naringin.

### Data Synthesis

**FIGURE 2:**
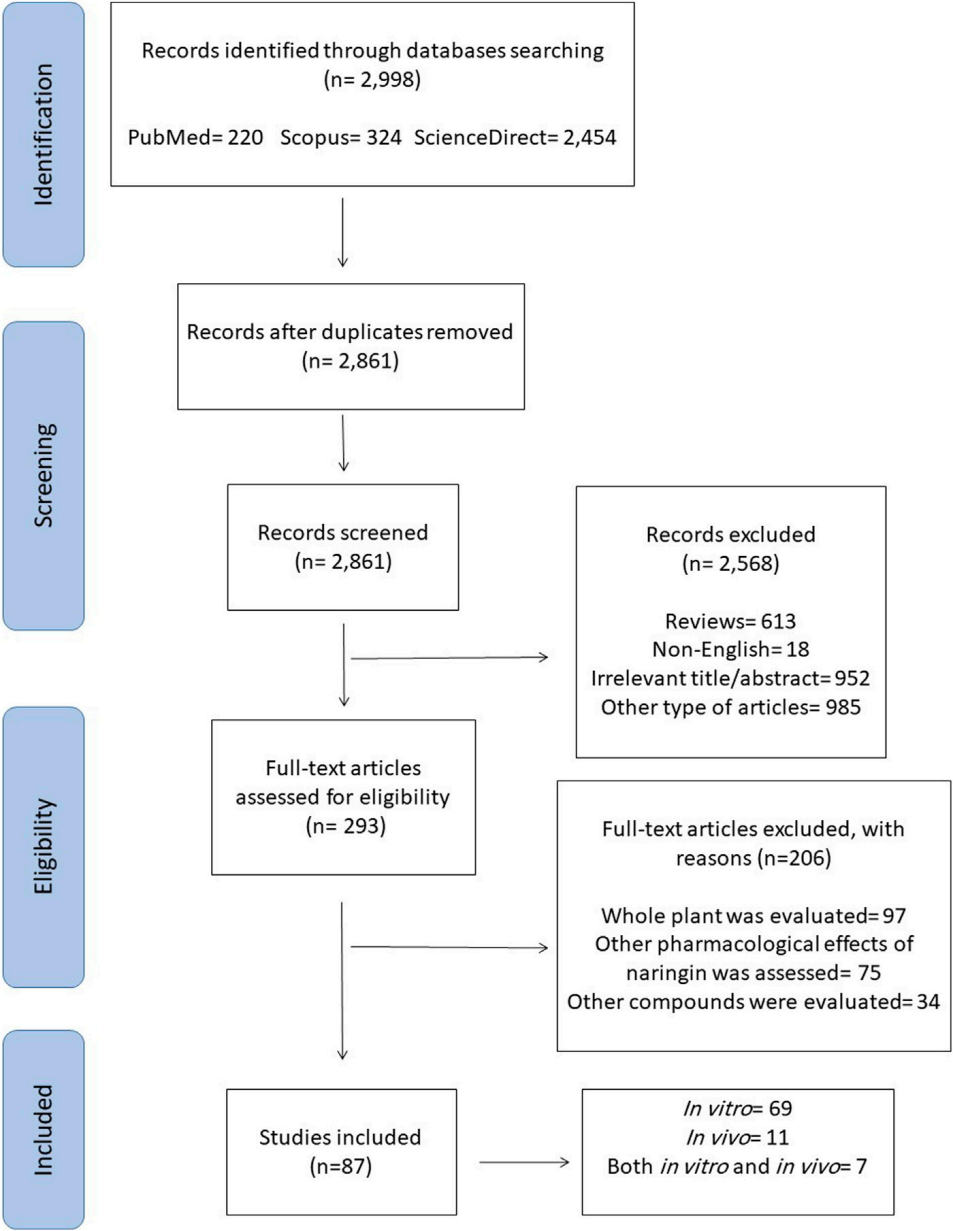
The PRISMA flow chart of the selection process for the included studies.

Finally, 87 articles were included in this study as demonstrated in a flowchart of the literature search and selection process (Figure 2). It was envisioned that studies would be too heterogeneous to be combined. Hence, a narrative synthesis was conducted. The results are summarized according to type of cancer and outcome measures assessed. The magnitudes of effects on each outcome measure are reported.

### Assessment of Bias and Errors

The primary search was conducted by two researchers, and they extracted the data independently, which limits the risk of bias and errors.

## Anticancer Activities of Naringin

Naringin has been shown to inhibit various cancers via different mechanisms, including growth suppression of malignant cells, apoptosis induction and cell cycle arrest, and modulation of oxidative stress, inflammation, and angiogenesis, through the regulation of several cellular signaling cascades. The antitumor effects and associated mechanisms of naringin in various cancers are presented in the following sections.

### Bladder Cancer

Bladder cancer includes a broad range of tumors, including transitional cell carcinoma, which is categorized into three types, namely superficial tumors, tumors confined to the bladder, and invasive tumors. In superficial bladder cancer, the risk of disease recurrence and/or progression to invasive diseases is high (Levi et al., 1993). For these cases, efficient preventive measures are required. One study determined a new mechanism of naringin anticancer activity observed in bladder cancer cell lines. Results demonstrated that naringin treatment suppressed cell viability and growth, and induced p21^WAF1^ expression and cell cycle arrest in 5,637 and T24 bladder carcinoma cell lines, potentially through suppressing the Ras/Raf/extracellular signal-regulated kinase (ERK)-signaling pathway (Table 3) (Kim et al., 2008). Another study showed that treatment with mononuclear palladium (II) complexes of naringin decreased the viability and proliferation of TCC bladder carcinoma cell lines, and these complexes noticeably showed major and selective cytotoxicity toward bladder cancer cells (Karami et al., 2018). In another *in vitro* study, naringin reduced cell proliferation and viability in TCC Human urinary bladder transitional cell carcinoma cells (Oršolić et al., 2009).

**TABLE 3:** Potential anticancer effects and related mechanisms of action of naringin based on *in vitro* studies.

Cancer type	Cell type	Conc	Source	Purity (%)	Quality control reported? (Y/N)	Duration	Anticancer effects	References
Bladder	T24 and 5,637 cell lines	50–150 μM	Wako pure chemical Industries, ltd. (Osaka, Japan)	ND	Y	24 h	↓Cell proliferation, ↓cell viability, ↓cell growth, ↑cell cycle arrest, ↑p21WAF1, ↑Ras, ↑Raf	Kim et al. (2008)
Bladder	TCC cell line	0.3–5 μM	Merck Chemical Co. (Darmstadt, Germany)	ND	Y	24–48 h	↓Cell proliferation, ↓cell viability	Karami et al. (2018)
Bladder	TCC cell line	75 μg/ml	Sigma-Aldrich (Munich, Germany)	ND	Y	24–72 h	↓Cell proliferation, ↓cell viability	Oršolić et al. (2009)
Blood (leukemia)	HL-60, Kasumi-1, and K562 cell lines	0.125–2 mg/ml	China Institute of drugs and Bioproducts (Beijing, China)	ND	Y-HPLC	24–48 h	↓Cell proliferation, ↑apoptosis, ↓Mcl-1	Dai et al. (2017)
Blood (leukemia)	U937 cell line	50–500 μM	Sigma-Aldrich (Lyon, France)	(90%)	Y	24 h	↓Cell proliferation, ↓cell growth, ↑cell death	Jin et al. (2009)
Blood (leukemia)	THP-1 cell line	50–400 μM	Gibco BRL (Gaithersburg, MD, United States)	ND	Y	48 h	↓Cell proliferation, ↓cell viability	Park et al. (2008)
Blood (leukemia)	HL-60 and THP-1 cell lines	40–80 μM	Sigma-Aldrich (Lyon, France)	ND	Y	6–24 h	No effect	Chen et al. (2003)
Blood (leukemia)	K562 cell line	5–500 μM	Quinabra Company (São José dos Campos, Brazil)	ND	Y	24–72 h	↓Cell number, ↓cell growth, ↑cell death, ↓DPPH	Pereira et al. (2007)
Blood (leukemia)	K562 cell line	1–100 μM	Fluka chemie GmbH (Buchs, Switzerland)	(≥95%)	Y- TLC and HPLC	20–100 h	↓VEGF	Mellou et al. (2006)
Blood (lymphoma)	P-388D1, L-1210 cell lines	1–2 mM	Sigma-Aldrich (St. Louis, MO, USA)	ND	Y	12 h	↑Cytotoxic activity, ↑anti-platelet aggregation activity, ↑trypsin inhibition	Kim et al. (1998)
Blood (lymphoma)	Raji cell line	10–1,000 μM	Extrasynthese-Genay (Lyon, France)	ND	Y	24 h	↓Cell proliferation, ↓cell growth	Ramanathan et al. (1992)
Brain	U-87cell line	5–30 μM	Sigma-Aldrich (Lyon, France)	(98%)	Y	24–48 h	↓Cell proliferation, ↓cell viability, ↓cell invasion, ↓tubulogenesis	Aroui et al. (2020)
Brain	U87 and U251 cell lines	10–40 μM	Invitrogen (Carlsbad, CA, USA)	ND	Y	12–48 h	↓Cell proliferation, ↓FAK/cyclin D1 pathway, ↑apoptosis, ↓cell invasion, ↓metastasis, ↓migration, ↓FAK/MMPs pathway, ↓kinase activity of FAK	Li et al. (2017)
Brain	U373 and U87 cell lines	5–100 μM	Sigma-Aldrich (Lyon, France)	ND	Y	12–24 h	↓Cell growth, ↓cell viability, ↓migration, ↓cell invasion, ↓MMP-9, ↓MMP-2, ↑MAPK signaling pathways, ↓metastasis	Aroui et al. (2016a)
Brain	U251 cell line	5–60 μM	Sigma-Aldrich (Lyon, France)	(98%)	Y	24 h	↓Cell proliferation, ↓cell viability, ↓cell invasion, ↓migration, ↓ MMP-9, ↓MMP-2, ↑TIMP-2, ↑TIMP-1, ↓p38 signal transduction pathways	Aroui et al. (2016b)
Brain (Glioma)	U343 and U118 cell lines	0.1–100 μM	Sigma-Aldrich (Steinheim, Germany)	ND	Y	24 h	↓VEGF	Schindler and Mentlein (2006)
Breast	MCF-7 cell line	50–400 μg/ml	Sigma-Aldrich (Berlin, Germany)	ND	Y-HPLC	48–72 h	↓Cell proliferation, ↓cell growth, ↑apoptosis	Elansary et al. (2020)
Breast	MCF7 cell line	5 μM	Sigma-Aldrich (St. Louis, MO, USA)	ND	Y-HPLC	12–48 h	↓Cell proliferation, ↓cell viability	Puranik et al. (2019)
Breast	MCF7 and HCT116 cell lines	0.78–100 μg/ml	Purified by Basta et al., 2020	ND	Y-TLC	48 h	↓Cell proliferation, ↓cell viability	Basta et al. (2020)
Breast	MCF7 cell line	0.78–100 μg/ml	Purified by Atta et al., 2019	ND	Y-TLC	48 h	↓Cell viability, ↓cell growth, ↑apoptosis	Atta et al. (2019)
Breast	MCF-7 cell line	200 μM	Sigma-Aldrich (St. Louis, MO, United States)	(≥95%)	Y	72 h	↓Cell proliferation, ↓cell viability, ↑apoptosis	Fazary et al. (2017)
Breast	MCF7 cell line	0.3–5 μM	Merck Chemical Co. (Darmstadt, Germany)	ND	Y	24–48 h	↓Cell proliferation, ↓cell viability	Karami et al. (2018)
Breast	MCF7 cell line	20–100 μM	Sigma-Aldrich (St. Louis, MO, USA)	(97%)	Y-HPLC	1–48 h	↓Cell proliferation, ↓cell viability	Selvaraj et al. (2014)
Breast	MCF-7 and MDA-MB-231 cell lines	5–100 μM	Sigma-Aldrich (Poznań, Poland)	ND	Y	24–48 h	↓Cell viability, ↑cell cycle arrest, ↑apoptosis	Kabała-Dzik et al. (2018)
Breast	CMT-U27 cell line	20–1,000 μM	Sigma-Aldrich Chemical Co. (Steinheim, Germany)	ND	Y	48 h	↓Cell proliferation, ↓cell viability	Özyürek et al. (2014)
Breast	MDA-MB-231, MDA-MB-468, and BT-549 cell lines	50–200 μM	Sigma-Aldrich (St. Louis, MO, USA)	(≥95%)	Y-HPLC	24–48 h	↓Cell proliferation, ↓cell growth, ↑cell cycle arrest, ↓cell viability, ↑apoptosis, ↓β-catenin pathway	Li et al. (2013a)
Breast	Ehrlich ascites tumor cells	5–100 μM	Sigma-Aldrich (St. Louis, MO, USA)	ND	Y	3–24 h	↑Tumor cell death, ↓tumor cell growth	Menon et al. (1995)
Breast	MDA-MB-231 cell line	0.1–100 μM	Sigma-Aldrich (St. Louis, MO, USA)	ND	Y	24 h	↓VEGF	Schindler and Mentlein (2006)
Cervical	C33A, SiHa, and HeLa cell lines	10–10,000 μM	Sigma-Aldrich (St. Louis, MO, USA)	(≥95%)	Y-HPLC	24 h	↓Cell viability, ↑cell cycle arrest, ↑apoptosis, ↓Wnt/β-catenin pathway	Chen et al. (2020)
Cervical	SiHa cell line	250–2000 μM	Sigma–Aldrich (St. Louis, MO, United States)	ND	Y	24–48 h	↓Cell proliferation, ↓cell viability, ↑cell cycle arrest, ↑apoptosis, ↑caspases, ↑p53, ↑Bax, ↑Fas	Ramesh and Alshatwi (2013)
Cervical	HeLa cell line	200–2000 μM	Sigma-Aldrich (St. Louis, MO, United States)	ND	Y	24 h	↓Cell proliferation, ↓cell growth, ↑apoptosis	Liu et al. (2017)
Cervical	HeLa cell line	200–3200 μM	Nacalai tesque (Kyoto, Japan)	ND	Y	48 h	↓Cell growth, ↑apoptosis, ↓NEU3, ↑EGFR/ERK signaling	Yoshinaga et al. (2016)
Cervical	HeLa cell line	10–1,000 μM	Extrasynthese-Genay (Lyon, France)	ND	Y	24 h	↓Cell proliferation, ↓cell growth	Ramanathan et al. (1992)
Cervical	HeLa cell line	50–400 μg/ml	Sigma-Aldrich (Berlin, Germany)	ND	Y-HPLC	48–72 h	↓Cell proliferation, ↓cell growth, ↑apoptosis	Elansary et al. (2020)
Cervical	HeLa cell line	200–1,500 μmol/L	Sigma-Aldrich (St. Louis, MO, United States)	ND	Y	3–48 h	↓Cell viability, ↓cell growth, ↑apoptosis, ↓NF-κB/COX-2-caspase-1 pathway	Zeng et al. (2014)
Colon	HT-29 cell line	50–400 μg/ml	Sigma-Aldrich (Berlin, Germany)	ND	Y-HPLC	48–72 h	↓Cell proliferation, ↓cell growth, ↑apoptosis	Elansary et al. (2020)
Colon	CT26 cell line	1–100 μg/ml	Purified by Zhou et al., 2018	ND	Y- HPLC		↓Cell proliferation, ↓cell viability, ↑apoptosis	Zhou et al. (2018)
Colon	SW480 cell line	12.5–200 μM	Sigma-Aldrich (St. Louis, MO, USA)	ND	Y	12–48 h	↓Cell proliferation, ↓cell viability	Chidambara Murthy et al. (2012)
Colorectal	HCT116 and SW620 cell lines	6–25 μg/ml	Beijing Solarbio Science and Technology Co., Ltd (Beijing, China)	ND	Y	12–72 h	↓Cell proliferation, ↑apoptosis, ↓PI3k/Akt/mTOR pathway	Cheng et al. (2020)
Colon	Colo 205 and Colo 320 cell lines	4–10 μg/ml	Purified by Ugocsai et al. (2005)	ND	Y	24 h	↑Apoptosis	Ugocsai et al. (2005)
Colon	COLO 320HSR, COLO 205, and HT 29 cell lines	200 μM	Sigma-Aldrich (St. Louis, MO, USA)	ND	Y	24 h	No effect	Shen et al. (2004)
Colon	HT29 cell line	10–250 μg/ml	Sigma-Aldrich (St. Louis, MO, USA)	ND	Y-HPLC and mass spectrometer	24–48 h	↓Cell proliferation, ↓cell growth	Ferreira et al. (2013)
Colon	HCT116 cell line	200 μM	Sigma Aldrich (St. Louis, MO, United States)	(≥95%)	Y	72 h	↓Cell proliferation, ↓cell viability, ↑apoptosis	Fazary et al. (2017)
Colon	HCT116 cell line	0.78–100 μg/ml	Purified by Basta et al., 2020	ND	Y-TLC	48 h	↓Cell proliferation, ↓cell viability	Basta et al. (2020)
Colon	SNU-C4 cell line	1–2 mM	Sigma-Aldrich (St. Louis, MO, USA)	ND	Y	12 h	↑Cytotoxic activity, ↑anti-platelet aggregation activity, ↑trypsin inhibition	Kim et al. (1998)
Colorectal	Caco-2 cell line	10–1,000 μM	Sigma-Aldrich (St. Louis, MO, USA)	ND	Y	24 h	↓Cell proliferation, ↓cell growth, ↓cell viability ↓GLO-I activity	Yadav et al. (2016)
Colon	HT-29 and Caco-2 cell lines	10–60 μM	Fluka Chemika-BioChemika (New York, USA)	(>95%)	Y	24–48 h	No effect	Kuo (1996)
Esophageal	YM1 cell line	300 μM	Sigma-Aldrich (St. Louis, MO, USA)	ND	Y	24 h	↓Cell proliferation, ↓cell viability	Tajaldini et al. (2020)
Head and Neck (laryngeal)	HEp2 cell line	3.8–500 μM	Sigma-Aldrich (St. Louis, MO, USA)	ND	Y	72 h	↓Cell viability, ↓lipid peroxidation, ↑CYP1A1	Durgo et al. (2007)
Liver	HepG2 cell line	12.5 μM–3.2 mM	Sigma–Aldrich (St. Louis, MO, United States)	(≥95%)	Y-HPLC	48 h	↓Cell viability, ↓cell growth, ↑apoptosis	Elsawy et al. (2020)
Liver	HepG2 cell line	5 μM	Sigma-Aldrich (St. Louis, MO, United States)	ND	Y	24 h	↓Cell proliferation, ↓cell viability	Syed et al. (2020)
Liver	HepG2 cell line	10–40 μM	Sigma-Aldrich (St. Louis, MO, USA)	ND	Y	24–72 h	↓Cell proliferation, ↑apoptosis, ↑Bax, ↓Bcl-2, ↑miR-19b	Xie et al. (2017)
Liver	HepG2 cell line	50–250 μg/ml	Sigma-Aldrich (St. Louis, MO, United States)	ND	Y	24 h	↓Cell proliferation, ↓cell viability, ↓cell growth, ↑apoptosis	Banjerdpongchai et al. (2016a)
Liver	HepG2 cell line	100 μg/ml	Sigma-Aldrich (St. Louis, MO, United States)	ND	Y	24 h	↓Cell proliferation, ↑apoptosis, ↑Bax, ↑Bak, ↓Bcl-xL, ↑tBid	Banjerdpongchai et al. (2016b)
Liver	HepG2 cell line	1–100 μg/ml	Purified by Zhou et al., 2018	ND	Y- HPLC		↓Cell proliferation, ↓cell viability, ↑apoptosis	Zhou et al. (2018)
Liver	HepG2, Huh-7, and HA22T cell lines	25–100 μM	Sigma–Aldrich (St. Louis, MO, United States)	(>98%)	Y	8–24 h	↓Cell invasion, ↓migration, ↓metastasis, ↓MMP-9, ↓PI3K/Akt, ↓MAPK, ↓IκB	Yen et al. (2015)
Liver	HepG2 cell line	10–250 μg/ml	Sigma-Aldrich (St. Louis, MO, USA)	ND	Y-HPLC and mass spectrometer	24–48 h	↓Cell proliferation, ↓cell growth	Ferreira et al. (2013)
Liver	HepG2, MCF-7, and HCT116 cell lines	200 mM	Sigma-Aldrich (St. Louis, MO, United States)	(≥95%)	Y	72 h	↓Cell proliferation, ↓cell viability, ↑apoptosis	Fazary et al. (2017)
Liver	HA22T and SK-Hep1 cell lines	10–100 μM	Aldrich chem. Co. (Milwaukee, WI, United States)	ND	Y	24 h	↓Cell viability, ↓cell growth	Hsiao et al. (2007a)
Liver	HepG2 cell line	1–2 mM	Sigma-Aldrich (St. Louis, MO, USA)	ND	Y	12 h	↑Cytotoxic activity, ↑anti-platelet aggregation activity, ↑trypsin inhibition	Kim et al. (1998)
Liver	Hepa-1c1c7 cell line	50–100 μM	Sigma-Aldrich (St. Louis, MO, USA)	ND	Y	72 h	No effect	Campbell et al. (2006)
Lung	A549 cell line	3–1,000 μM	Purified by Nie et al., 2012	(>98.3%)	Y-determined by peak area normalization	24–96 h	↓Cell proliferation, ↓cell viability	Nie et al. (2012)
Lung	A549 and LLC cell lines	10–100 μM	Aldrich chem. Co. (Milwaukee, WI, United States)	ND	Y	24 h	↓Cell viability, ↓cell growth	Hsiao et al. (2007a)
Lung	H69AR cell line	6–25 μg/ml	ND	ND	ND	24 h	↓Cell proliferation, ↓cell growth, ↑apoptosis, ↑miR-126, ↓PI3K, ↓p-Akt, ↓p-mTOR, ↓VCAM-1, ↓NF-κB, ↓PI3K/Akt/mTOR pathway	Chen et al. (2018)
Lung	A549 cell line	10–50 μM	Aldrich chem. Co. (Milwaukee, WI, United States)	ND	Y	24 h	↓Cell viability, ↓cell invasion, ↓cell-matrix adhesion, ↓cellular motility	Hsiao et al. (2007b)
Lung	A549 cell line	1–2 mM	Sigma-Aldrich (St. Louis, MO, USA)	ND	Y	12 h	↑Cytotoxic activity, ↑anti-platelet aggregation activity, ↑trypsin inhibition	Kim et al. (1998)
Lung	A549 cell line	10–120 μg/ml	Sigma-Aldrich, (St. Louis, MO, United States)	ND	Y	6–24 h	↓Cell proliferation, ↓cell viability, ↑apoptosis	Garcia et al. (2019)
Lung	A549 cell line	0.78–100 μg/ml	Purified by Atta et al., 2019	ND	Y-TLC	48 h	↓Cell viability, ↓cell growth, ↑apoptosis	Atta et al. (2019)
Lung	HeLa and A549 cell lines	200–3200 μM	Nacalai Tesque, Inc. (Kyoto, Japan)	ND	Y	48 h	↓Cell growth, ↑apoptosis, ↓NEU3, ↑EGFR/ERK signaling	Yoshinaga et al. (2016)
Neuroblastoma	SH-SY5Y cell line	1–10 μM	Sigma-Aldrich (St. Louis, MO, USA)	ND	Y	24 h	↓Cell viability, ↑cell death	Kim et al. (2009)
Ovarian	SKOV3/CDDP cell line	10–40 μmol/L	Institute of pharmacology at Nanchang university (Nanchang, China)	ND	Y	48 h	↓NF-κB, ↓P-gp	Zhu et al. (2018)
Ovarian	SKOV3/CDDP cell line	10–40 μmol/L	Shandong Qilu Pharmaceutical Co., Ltd. (Shandong, China)	ND	Y	48 h	↓NF-κB, ↓COX-2	Zhu et al. (2017)
Ovarian	OVCAR-3 cell line	5–160 μM	Sigma-Aldrich (St. Louis, MO, USA)	ND	Y	24 h	No effect	Luo et al. (2008)
Prostate	PC-3 and LNCaP cell lines	2.5–300 μM	Selleck (Maple Valley, WA, USA)	ND	Y	24–48 h	↓Cell growth, ↓migration, ↓cell invasion, ↑apoptosis, ↑Bax, ↓p-↓STAT3, ↓survivin, ↓Bcl-2, ↓p-Akt	Wu et al. (2019)
Prostate	PC3, DU145, and LNCaP cell lines	3.9–500 μM	Sigma-Aldrich (St. Louis, MO, United States)	ND	Y	72 h	↓Cell survival, ↓cell viability, ↑apoptosis, ↑cell cycle arrest, ↑PTEN, ↓nuclear factor-κB p50 protein, ↓cell migration, ↓NF-κB signaling	Erdogan et al. (2018)
Prostate	DU145 cell line	50–250 μM	Sigma-Aldrich (Poznan, Poland)	ND	Y	24 h	↓Cell proliferation, ↓cell viability, ↓cell number, ↑oxidative stress, ↑apoptosis	Lewinska et al. (2015)
Sarcoma (osteosarcoma)	MG63 and U2OS cell lines	10–20 μmol/L	Beyotime Biotechnology (Shanghai, China)	ND	Y	24 h	↓Cell proliferation, ↓cell invasion, ↑apoptosis, ↓Zeb1, ↓cell migration, ↑cell cycle arrest	Ming et al. (2018)
Sarcoma (osteosarcoma)	MG-63 cell line	1–100 μg/ml	Purified by Zhang et al., 2018a	ND	Y-HPLC and Mass spectrometry	24–72 h	No effect	Zhang et al. (2018a)
Sarcoma (chondrosarcoma)	JJ012 cell line	3–30 μM	Sigma-Aldrich (St. Louis, MO, United States)	ND	Y	24–48 h	↓Cell invasion, ↓migration, ↓VCAM-1, ↑miR-126	Tan et al. (2014)
Skin (Melanoma)	A375 and A875 cell lines	10–40 μM	ND	ND	ND	12–60 h	↓Cell proliferation, ↓cancer metabolism, ↑cell cycle arrest, ↑apoptosis, ↓cell growth, ↓cell invasion, ↓migration, ↓c-Src	Guo et al. (2016)
Skin (Melanoma)	MO4 cell line	0.5 mM	Provided by dr. J, A. Attaway (department of citrus, state of Florida, United States)	ND	Y- Reversed-phase high-pressure liquid chromatography	4 days	↓Invasion	Bracke et al. (1991)
Skin (Melanoma)	B16F10 cell line	5–500 μM	Quinabra Company (São José dos Campos, Brazil)	ND	Y	24–72 h	↓Cell number, ↓cell growth, ↑cell death, ↓DPPH	Pereira et al. (2007)
Stomach (Gastric)	AGS cell line	1–3 mM	Sigma-Aldrich (St. Louis, MO, United States)	ND	Y	3–24 h	↑ROS, ↑ERK1/2-p38 MAPKs, ↑autophagy cell death	Raha et al. (2020)
Stomach (Gastric)	AGS cell line	10–100 μM	Aldrich Chemical Co. (Milwakee, WI, United States)	ND	Y	24 h	↓Cell viability, ↓cell growth	Hsiao et al. (2007a)
Stomach (Gastric)	AGS cell line	1–3 mM	Sigma-Aldrich (St. Louis, MO, United States)	ND	Y	24–48 h	↓Cell proliferation, ↓cell growth, ↓PI3K/Akt/mTOR, ↑MAPKs, ↑p21^CIPI/WAFI^, ↑autophagosome	Raha et al. (2015)
Stomach (Gastric)	SNU-1	1–2 mM	Sigma-Aldrich (St. Louis, MO, USA)	ND	Y	12 h	↑Cytotoxic activity, ↑anti-platelet aggregation activity, ↑trypsin inhibition	Kim et al. (1998)
Thyroid	TPC-1 and SW1736 cell lines	6–25 μg/ml	Beyotime Biotechnology (Shanghai, China)	ND	Y	24–72 h	↓Cell proliferation, ↑apoptosis, ↓PI3k/Akt pathway	Zhou et al. (2019)

**Note:** A down arrow indicates a reduction or decrease and an up arrow indicates an increase. Bak, Bcl-2 homologous antagonist/killer; Bax, Bcl-2-associated X protein; Bcl-2, B-cell lymphoma 2; Bcl-xL, B-cell lymphoma-extra-large; COX-2, cyclooxygenase- 2; c-Src, proto-oncogene tyrosine-protein kinase Src; DPPH, diphenylpicrylhydrazyl radical; EGFR, epidermal growth factor receptor; ERK, extracellular signal-regulated kinase; FAK, focal adhesion kinase; GLO-I, glyoxalase-I; IκB, inhibitor of NF-κB; MMPs, matrix metallopeptidases; MAPK, mitogen-activated protein kinase; Mcl-1, myeloid cell leukemia 1; miR, microRNA; mTOR, mammalian target of rapamycin; ND, not determined; NEU3, plasma membrane-associated sialidase; NF-κB, nuclear factor-κB; p53, tumor protein p53; p38 MAPKs, p38 mitogen-activated protein kinases; p-Akt, phosphorylated Akt; P-gp, P-glycoprotein; PI3K, phosphatidylinositol-3-kinase; p-mTOR, phosphorylated mammalian target of rapamycin; p-STAT3, phosphorylated signal transducer and activator of transcription 3; ROS, reactive oxygen species; tBid, truncated BH3 interacting domain death agonist; TIMP, tissue inhibitor of metalloproteinase; VCAM-1, vascular cell adhesion molecule 1; VEGF, vascular endothelial growth factor; Zeb1, zinc finger E-box binding homeobox 1.

### Blood Cancer

Leukemia is one form of the hematological malignancies with particularly high mortality (Vardiman et al., 2009). At present, leukemia treatment relies on chemotherapies to abrogate malignant cells or to promote differentiation in leukemia cells. Conversely, the available chemotherapies commonly have severe adverse effect (Goldman and Melo, 2003). Accordingly, the discovery of novel therapeutic reagents with a magnificently safe profile is required. One study indicated that treatment of K562, HL-60, and Kasumi-1 human myeloid leukemia cells with naringin blocked cell proliferation and promoted their apoptosis in a time- and concentration-dependent way, via downregulation of Mcl-1 expression and activation of caspase and PARP pathway (Dai et al., 2017). Naringin treatment also enhanced cell death and decreased cell proliferation and growth in U937 (Jin et al., 2009) and THP-1 (Park et al., 2008) human leukemia cells. However, one study showed that naringin had no cytotoxic effect on THP-1 and HL-60 are leukemia cell lines (Chen et al., 2003). In another study, a naringin-derived copper (II) complex 1 was engineered, and its anticancer effect was investigated. Results showed that treatment of K562 human chronic myeloid leukemia cells with naringin–Cu (II) complex 1 or naringin reduced cell proliferation and growth, increased cell death, and decreased diphenylpicrylhydrazyl radical (DPPH). Results also showed that naringin–Cu (II) complex 1 had greater anti-inflammatory, antioxidant, and anticancer activities in comparison to free naringin without decreasing cell viability (Pereira et al., 2007). One study demonstrated that *in vitro* naringin treatment reduced the VEGF production in K562 human leukemia cells (Mellou et al., 2006). Naringin also increased the inhibitory activity of trypsin and enhanced the antiplatelet aggregation activity and cytotoxic activity against P-388D1 (mouse lymphoid neoplasma) and L-1210 (mouse lymphocytic leukemia) cell lines (Kim et al., 1998). Another study also showed that *in vitro* naringin treatment suppressed cell proliferation and growth in Raji lymphoma cells (Ramanathan et al., 1992).

### Brain Cancer

Gliomas are the most aggressive and frequent brain tumors, and regardless of progress in therapeutic management, they are still lethal. Accordingly, various therapeutic approaches are required to treat this aggressive disease (Martínez-Vélez et al., 2018). One study examined the antitumor effects of naringin treatment *in vitro* and *in vivo*. Results indicated that naringin had a toxic impact on the U-87 cell line and reduced cancer cell proliferation and viability in a concentration-dependent way. Moreover, naringin administration also suppressed tubulogenesis and angiogenesis and reduced tumor size and cell invasion U-87 mouse xenograft tumor model (Table 4) (Aroui et al., 2020). Another study demonstrated that naringin could specifically suppress the focal adhesion kinase (FAK) activity and inhibit the FAKp-Try397 and its downstream pathway in glioblastoma cells. Treatment of U87 and U251 glioblastoma cells with naringin blocked cell proliferation through suppression of the FAK/cyclin D1 pathway and induction of cell apoptosis via the FAK/Bad pathway. It also inhibited cell metastasis and invasion by suppressing the FAK/MMPs pathway (Li et al., 2017). Another study demonstrated that naringin had inhibitory impacts on the migration, adhesion, and invasion of U87 and U373 human glioblastoma cells in a concentration-dependent way. Additionally, naringin inhibited several aspects of the MAPK signaling pathways, including p38, ERK, and JNK, and led to the downregulation of the MMP-9 and MMP-2 expression and enzymatic activities, contributing to the suppression of metastasis of glioblastoma cells (Aroui et al., 2016a). Treatment with naringin also reduced cell proliferation and viability in U251 glioma cells. Additionally, naringin suppressed cell invasion and migration via the modulation of matrix metallopeptidase-9 (MMP-9) and MMP-2 expressions. Therefore, naringin might have therapeutic potential for the control of the invasiveness of malignant gliomas through suppressing the p38 signal transduction pathways (Aroui et al., 2016b). In an *in vitro* study, the treatment of U343 and U118 glioma cells with naringin showed a reduction in VEGF levels (Schindler and Mentlein, 2006). These findings have shown a new potential for exploring the capability of naringin as a promising therapeutic agent in gliomas.

**TABLE 4:** Potential anticancer effects and related mechanisms of action of naringin based on *in vivo* studies.

Cancer type	Animal model	Dose	Source	Purity (%)	Quality control reported? (Y/N)	Duration	Anticancer effects	References
Brain	Athymic mice bearing U-87 tumor	60–180 mg/kg	Sigma-Aldrich (St. Louis, MO, USA)	(98%)	Y	7 days	↓Tumor size, ↓survival, ↓angiogenesis	Aroui et al. (2020)
Breast	SCID female mice bearing MDA-MB-231 tumor	100 mg/kg	Sigma-Aldrich (St. Louis, MO, USA)	(≥95%)	Y-HPLC	1–5 weeks	↓Tumor volume, ↓tumor weight	Li et al. (2013a)
Breast	Swiss albino mice bearing ehrlich ascites tumor cells	100 mg/kg	Sigma-Aldrich Chemie GmbH (Munich, Germany)	ND	Y	90 days	↑Survival, ↓tumor growth, ↓tumor cell proliferation, ↑tumor regression	Benkovic et al. (2007)
Breast	Swiss albino mice bearing ehrlich ascites tumor cells	100 mg/kg	Sigma–Aldrich chemie GmbH (Germany)	ND	Y	5 days	↓Tumor growth, ↑survival, ↓tumor cell proliferation	Oršolić et al. (2010)
Breast	Swiss albino mice bearing ehrlich ascites tumor cells	100 mg/kg	Sigma-Aldrich chemie GmbH (Munich, Germany)	ND	Y	5 days	↓Tumor growth, ↑survival, ↓tumor cell proliferation	Knežević et al. (2011)
Breast	Female Sprague-dawley rats with DMBA-induced breast tumors	500 mg/100 g diet	Provided by dr. W. Widmer (state of Florida dept. of citrus, United States)	ND	N	10 weeks	↓Tumor development, ↓tumor weight	So et al. (1996)
Cervix	Female athymic nude mice bearing HeLa tumors	20 mg/kg	Sigma-Aldrich (St. Louis, MO, United States)	ND	Y	20 days	↓Tumor growth, ↑apoptosis	Liu et al. (2017)
Colon	Male C57BL/6 mice with AOM/DSS-induced colon carcinogenesis	50–100 mg/kg	Sigma-Aldrich (St. Louis, MO, USA)	(≥98%)	Y	1–63 days	↓Tumor growth, ↓tumor size, ↓STAT3, ↓p- mTOR, ↓NF-κB	Zhang et al. (2018b)
Colon	Male Sprague–Dawley rats with AOM-induced colon carcinogenesis	200 mg/kg	Purified by Vanamala et al., 2006	ND	Y-Reverse phase liquid chromatography	10 weeks	↓Tumor cell proliferation, ↑apoptosis	Vanamala et al. (2006)
Esophagus	Male nude mice with YM1 xenograft tumors	50 mg/kg	Sigma-Aldrich (St. Louis, MO, USA)	ND	Y	14 days	↓Tumor size, ↓tumor growth	Tajaldini et al. (2020)
Head and neck (oral cavity)	Hamster cheek pouch oral cancer model	0.5–8.0 mg/kg	ND	ND	ND		↓Tumor growth, ↓tumor number, ↓tumor burden	Miller et al. (2008)
Liver	Male Wistar rats bearing DEN-induced hepatocellular carcinoma	40 mg/kg	Sigma-Aldrich (St. Louis, MO, USA)	ND	Y	6–16 weeks	↓Cell proliferation, ↑apoptosis, ↓AgNOR/nuclei	Thangavel and Vaiyapuri (2013)
Ovary	Female BALB/c nude mice bearing SKOV3 tumor	0.5–2 mg/kg	Merck KGaA (Darmstadt, Germany)	(>90%)	Y	10 days	↑Apoptosis, ↓tumor size, ↓tumor weight, ↓tumor growth, ↓Bcl-xL, ↓Bcl-2, ↓cyclin D1, ↓c-Myc, ↓ survivin, ↑caspase-3, ↑caspase-7	Cai et al. (2018)
Prostate	Male SCID mice bearing PC-3 and LNCaP tumors	50 mg/kg	Selleck (Maple Valley, WA, USA)	ND	Y	16 days	↓Tumors growth, ↑apoptosis	Wu et al. (2019)
Sarcoma (osteosarcoma)	Female, athymic nude BALB/c mice bearing MG63 tumor	5–10 mg/kg	Beyotime Biotechnology (Shanghai, China)	ND	Y	16 days	↓Invasion, ↓migration ↓cyclin D1, ↓MMP-2, ↓Bcl-2, ↓Zeb1	Ming et al. (2018)
Sarcoma	Male ddY mice bearing S180 tumor	30–300 mg/kg	Sigma-Aldrich (St. Louis, MO, USA)	ND	Y	5 days	↓Tumor growth	Kanno et al. (2005)
Sarcoma (carcinosarcoma)	Male Wistar rats bearing W256 tumor	10–35 mg/kg	Sigma-Aldrich (St. Louis, MO, USA)	ND	Y	50 days	↓Tumor growth, ↑survival, ↓TNF-α, ↓IL-6	Camargo et al. (2012)
Skin (melanoma)	C57BL/6 female mice bearing B16FlO tumors	200 nmol/kg	Sigma-Aldrich (St. Louis, MO, USA)	ND	Y	10 days	↓Metastatic foci formation, ↑survival, ↓lung tumor nodules	Menon et al. (1995)

**Note:** A down arrow indicates a reduction or decrease and an up arrow indicates an increase. AgNOR, argyrophilic nucleolar organizer region; Bcl-2, B-cell lymphoma 2; Bcl-xL, B-cell lymphoma-extra-large; DEN, diethylnitrosamine, DMBA, 7,12-dimethylbenz[a]anthracene; MMP-2, matrix metallopeptidase-2; mTOR, mammalian target of rapamycin; ND, not determined; NF-κB, nuclear factor-κB; STAT3, signal transducer and activator of transcription three; Zeb1, zinc finger E-box binding homeobox 1.

### Breast Cancer

Breast cancer is a heterogeneous group of tumors. Lately, a large number of personalized treatments for breast cancer have been introduced, with proven effectiveness (Cadoo et al., 2013). Natural products containing bioactive compounds are being used for both chemotherapy and cancer chemoprevention. Treatment with naringin suppressed proliferation and growth, and also increased apoptosis in MCF-7 cell lines (Puranik et al., 2019; Elansary et al., 2020). One study indicated that treatment with naringin alone or in combination with the sodium salt of carboxymethyl cellulose-phenyl alanine and sodium caseinate-phenyl alanine reduced viability and proliferation of MCF-7 cell line. Besides, naringin in hybrids had a greater cytotoxic effect in comparison to naringin alone (Basta et al., 2020). Moreover, treatment with naringin and its metal complexes decreased cell viability and proliferation and increased apoptosis in MCF-7 cell line (Atta et al., 2019). Based on the results, metal complexes of naringin demonstrated the highest cytotoxicity against cancer cells in comparison with naringin alone (Fazary et al., 2017). In another study, mononuclear palladium (II) complexes of naringin were synthesized, and the cytotoxic effect against MCF-7 cells was investigated. Results showed that naringin complexes reduced the viability and proliferation of breast cancer cells (Karami et al., 2018). Another study showed that treatment with naringin and its iron and copper complexes resulted in a reduction in the proliferation and viability of MCF-7 breast adenocarcinoma cell line (Selvaraj et al., 2014). One study, using different breast cancer cells (MCF-7 and MDA-MB-231), indicated that naringin reduced cell viability and promoted apoptosis and cycle arrest in breast cancer cells (Kabała-Dzik et al., 2018).

Recently, there has been an increased interest in polyphenolic antioxidants because of their health advantages, which has resulted in the evaluation of novel polyphenolic compounds with increased antioxidant activity, such as naringin oxime. Treatment with naringin oxime reduced cell viability and proliferation in CMT-U27 canine mammary carcinoma cells. New oxime-type antioxidants, such as naringin oxime, can be synthesized from various flavanones, and these derivatives may be used as anticancer and radioprotective agents (Özyürek et al., 2014). In another study, triple-negative breast cancer cell lines (MDA-MB-231, MDA-MB-468, and BT-549) were used to investigate the antitumor effect and related mechanisms of naringin. Results showed that naringin suppressed cell proliferation and increased G1 cycle arrest and apoptosis, accompanied by enhanced p21 and reduced survivin. Besides, the β-catenin signaling pathway was blocked by naringin treatment. Correspondingly, the anticancer potential of naringin was investigated in an *in vivo* condition, and naringin decreased tumor volume and weight in naringin-treated MDA-MB-231 xenograft mice (Li et al., 2013a).

Irinotecan is a semi-synthetic derivate of camptothecin that belongs to the class of topoisomerase I inhibitors and has significant activity against various cancers. *In vivo* studies showed that treatment with naringin alone or combined with irinotecan suppressed tumor growth and tumor cell proliferation and promoted survival in Ehrlich ascites tumor cell bearing-mice. Besides, naringin enhanced irinotecan-induced cytotoxicity to cancer cells in mice bearing Ehrlich ascites tumors, while protecting normal cells (Benkovic et al., 2007; Oršolić et al., 2010; Knežević et al., 2011). Another study also showed that naringin increased cell death in Ehrlich ascites tumor cells (Menon et al., 1995). Naringin treatment also inhibited the development of mammary tumors and decreased the tumor weight in Sprague-Dawley rats induced by 7,12-dimethylbenz[a]anthracene (DMBA) (So et al., 1996). Numerous solid tumors induce vascular proliferation through the production of angiogenic factors, especially vascular endothelial growth factor (VEGF). One study demonstrated that naringin treatment decreased the level of secreted VEGF from MDA-MB-231 cells (Schindler and Mentlein, 2006).

### Cervical Cancer

Cervical cancer is the second-highest cause of death among women between the ages of 20 and 39 years (Fidler et al., 2017). Although chemotherapy is the standard therapeutic option, the survival rates of patients with cervical cancer are poor and need improvement via investigation of specific antitumor agents with less adverse effects on healthy cells (Pfaendler and Tewari, 2016). Hence, novel therapeutic targets are urgently required for the improvement of cervical cancer therapeutics. One study evaluated the antiproliferative effect and the associated mechanism of naringin-induced cell death in C33A, SiHa, and HeLa human cervical cancer cell lines. Results demonstrated that naringin treatment reduced cell viability and induced endoplasmic reticulum stress-mediated apoptosis. It also inhibited the Wnt/β-catenin pathway by reducing the protein expression and phosphorylation of glycogen synthase kinase-3β (Ser9) and β-catenin (Ser576), while simultaneously induced cell cycle arrest (Chen et al., 2020). *In vitro* treatment of SiHa cells with naringin decreased cell proliferation and viability by G2/M cell cycle arrest and induced apoptosis via DwM disruption, and intrinsic and extrinsic pathway activation (Ramesh and Alshatwi, 2013).

The doxorubicin (DOX) application in cervical cancer chemotherapy is severely hampered by the DOX side effects. The formation of DOX-iron complexes by oxygen free radicals plays an important role in DOX-induced toxicity (Myers, 1998). Fortunately, flavonoids have excellent radical scavenging and iron-chelating properties (Kaiserová et al., 2007), and they can act as an effective modulator for DOX-induced toxicity. Treatment with naringin, DOX, and their combination reduced cell proliferation in HeLa human cervical cancer cells and suppressed HeLa cervical tumor and induced cell apoptosis in tumor-bearing mice. More importantly, the combined treatment had a greater antitumor effect in comparison to either agent alone (Liu et al., 2017). Another study showed that naringin treatment suppressed plasma membrane-associated sialidase (NEU3), and the NEU3-inhibitory effect of naringin promoted GM3 accumulation in HeLa cells, resulting in epidermal growth factor receptor (EGFR)/ERK signaling attenuation accompanied by a reduction in cell growth and enhancement of apoptotic cells (Yoshinaga et al., 2016). Additional studies demonstrated that naringin decreased proliferation and viability and also induced apoptosis of HeLa cervical adenocarcinoma cells (Ramanathan et al., 1992; Elansary et al., 2020) by blocking the NF-κB/cyclooxygenase-2 (COX-2)-caspase-1 pathway (Zeng et al., 2014).

### Colon Cancer

Colorectal cancer (CRC) is one of the most frequent malignant tumors. The primary methods for CRC treatment are radiotherapy, chemotherapy, and surgery. However, because of the challenges rising from drug resistance, it is vital to explore additional effectual compounds targeting alternative signaling pathways (Van der Jeught et al., 2018). Studies show that naringin has antineoplastic activities and treatment with naringin can reduce proliferation, and viability, while also enhancing apoptosis in HT-29 colon adenocarcinoma (Elansary et al., 2020) and CT26 colorectal cancer cell lines (Zhou et al., 2018). Another study indicated that treatment of human colon tumor cell lines (HCT116 and SW620) with naringin suppressed the CRC cell viability and proliferation, and promoted apoptosis by suppressing the phosphoinositide-3 kinase (PI3K)/protein kinase-B (also known as Akt)/mammalian target of rapamycin (mTOR) signaling pathway (Chidambara Murthy et al., 2012; Cheng et al., 2020). Naringin treatment also induced apoptosis in Colo 205 and Colo 320 human colon cancer cells (Ugocsai et al., 2005). However, one study indicated that naringin had no inhibitory effect on cell growth of COLO 320HSR, COLO 205, and HT 29 colon cancer cells (Shen et al., 2004). In another study, naringin treatment interestingly abrogated cell growth and proliferation in human colon adenocarcinoma cells (HT29) in a concentration-dependent manner, and bio-transformed naringin showed significant antiproliferative activity (Ferreira et al., 2013). Also, treating HCT116 human colorectal carcinoma cells with naringin and synthesized binary and ternary platinum and vanadium metal complexes has shown moderate cytotoxic activities, with enhanced apoptosis and reduced cell viability and proliferation (Fazary et al., 2017). In another study, the antitumor effect of naringin was examined alone and in hybrids with SCMC-PA and SC-PA conjugates against the HCT116 colon cancer cells. Results showed that naringin, individually or in hybrids, reduced the proliferation and viability of colon cancer cell lines (Basta et al., 2020). Naringin also increased the inhibitory activity of trypsin and enhanced the antiplatelet aggregation activity and cytotoxic activity toward SNU-C4 human colon cancer cells (Kim et al., 1998).

In an *in vivo* study, the cytotoxic impacts of naringin on azoxymethane (AOM)/dextran sulfate sodium (DSS)-induced colorectal carcinogenesis and inflammation in C57BL/6 mice were investigated. Results indicated that naringin-treatment reduced tumor size and growth in C57BL/6 mice through inhibiting robust endoplasmic reticulum stress-induced autophagy in colorectal mucosal cells (Zhang et al., 2018b). Another study showed that naringin reduced tumor cell proliferation and promoted apoptosis in AOM-injected Sprague–Dawley rats (Vanamala et al., 2006). Glyoxalase-I (GLO-I), the ubiquitous detoxification system component, is involved in methylglyoxal (MG) conversion to D-lactate in the glycolytic pathway and has been shown to be regularly overexpressed in several cancer cells (Thornalley, 2008). One study showed that naringin reduced GLO-I activity and suppressed cell proliferation and viability in Caco-2 human epithelial colorectal adenocarcinoma cells (Yadav et al., 2016). However, naringin had no cytotoxic impact on Caco-2 and HT-29 human colon adenocarcinoma cells (Kuo, 1996).

### Esophageal/Head and Neck Cancer

Esophageal carcinoma is a relatively rare cancer with a high death rate worldwide (Pennathur et al., 2013). DOX is an important chemotherapy agent that has been widely used as an antitumor agent (Hajjaji et al., 2012). Recent works demonstrated that the combination of herbal medicines and chemotherapy drugs have several advantages. One study showed that treatment with naringin alone or combined with DOX reduced cell viability and proliferation in YM1 esophageal cancer cell line, and reduced tumor size in xenograft mice tumor model (Tajaldini et al., 2020). An *in vivo* study indicated that naringin treatment markedly reduced tumor size and growth, and also significantly decreased tumor burden in the hamster cheek pouch oral cancer model (Miller et al., 2008). A separate study demonstrated that *in vitro* treatment with naringin reduced cell viability in HEp2 human laryngeal carcinoma cells. Naringin also reduced lipid peroxidation and enhanced cytochrome P-450 (CYP) 1A1 expression in laryngeal cancer cell lines (Durgo et al., 2007).

### Liver Cancer

Irrespective of the combined efforts of researchers and clinicians around the world, there has been a continuous increase in the incidence rate of hepatocellular carcinoma (HCC) over the last two decades (Alwhibi et al., 2017). In one study, HepG2 cell lines were used to examine the possible antiproliferative and cytotoxic effects of naringin and/or methotrexate (MTX). Naringin and/or MTX treatment exhibited cytotoxic and antiproliferative effects and induced apoptosis in HepG2 hepatocellular cancer cells via Bax activation and downregulation of Bcl-2 protein expression in a concentration-dependent way. Additionally, naringin potentiated the viability and cytotoxic effect of MTX in HepG2 cells (Elsawy et al., 2020). In another study, naringin substantially reduced the viability and proliferation of HepG2 cells (Syed et al., 2020). Naringin treatment also significantly suppressed proliferation, upregulated the expression of microRNA (miR)-19b, and promoted apoptosis in HepG2 cells. Additionally, it downregulated the Bcl-2 protein expression and upregulated the Bax protein expression to trigger apoptosis (Xie et al., 2017). Naringin blocked proliferation and enhanced early apoptosis of HepG2 cells via activation of Bid proteolysis mediated by caspase-8 and caspase-9. Therefore, the intrinsic and extrinsic pathways were linked in naringin-mediated apoptosis in HepG2 cells. Additionally, increased expression levels of pro-apoptotic Bak and Bax proteins and reduced levels of anti-apoptotic Bcl-xL protein were demonstrated, verifying the participation of the mitochondrial pathway (Banjerdpongchai et al., 2016b). Treatment with naringin reduced proliferation, viability, and growth and promoted apoptosis in HepG2 cells through extrinsic and intrinsic pathways (Banjerdpongchai et al., 2016a; Zhou et al., 2018). In another *in vitro* study, the cytotoxic effect of naringin in different hepatocellular carcinoma cells (Huh-7, HepG2, and HA22T) was investigated. Treatment with naringin inhibited MMP-9 transcription by suppressing NF-κB and activator protein-1 (AP-1) activity. It inhibited 12-O-tetradecanoylphorbol 13-acetate (TPA)-induced AP-1 activity by suppressing the phosphorylation of the c-Jun N-terminal kinase (JNK) and ERK signaling cascades, and it inhibited TPA-induced suppression of NF-κB nuclear translocation by IκB. This data demonstrates that naringin inhibits the metastasis and invasion of HCC cells by suppressing multiple signal transduction pathways (Yen et al., 2015). In another study, treatment with various concentrations of naringin hampered cell growth and cell proliferation of HepG2 cells and biotransformation with tannase significantly increased its antiproliferative activity (Ferreira et al., 2013).

Diethylnitrosamine (DEN) is one of the major environmental carcinogens and hepatotoxins (Sreepriya and Bali, 2005). In an *in vivo* experiment, the apoptotic and antiproliferative effect of naringin on DEN-induced liver carcinogenesis in male Wistar rat models was evaluated. Results showed that treatment with naringin significantly reduced the levels of proliferating cell nuclear antigen (PCNA) and the argyrophilic nucleolar organizer region (AgNOR)/nuclei. Naringin also suppressed proliferation and enhanced apoptosis in the liver cancer cells of rats (Thangavel and Vaiyapuri, 2013). In one study, cytotoxic actions of the synthesized binary and ternary platinum and vanadium metal complexes of naringin were evaluated using HepG2 cells. Results demonstrated that treatment with naringin and its metal complexes reduced cell viability and proliferation, and enhanced apoptosis in liver cancer cells. Additionally, metal complexes of naringin had a greater cytotoxic effect compared with naringin alone (Fazary et al., 2017). Moreover, naringin treatment of Hep1 and HA22T human liver cancer cells resulted in the suppression of cell viability and growth (Hsiao et al., 2007a). In another study, naringin treatment increased cytotoxic and antiplatelet aggregation activities and enhanced trypsin inhibition in HepG2 cells (Kim et al., 1998). However, at least one study demonstrated that naringin did not suppress cell growth in Hepa-1c1c7 mouse liver cancer cell line (Campbell et al., 2006).

### Lung Cancer

Lung carcinoma is one of the main causes of cancer-related death worldwide (Jemal et al., 2002). The high death rate of lung cancer is possibly due to challenges associated with diagnosis and a high metastatic potential (Sangodkar et al., 2010). Consequently, it is essential to determine non-toxic alternative therapies to improve the responsiveness of lung cancers to chemotherapy. Treatment with naringin reduced viability and growth in the A549 human lung adenocarcinoma (Nie et al., 2012), and Lewis lung carcinoma (LLC) cell lines (Hsiao et al., 2007a). Naringin treatment also suppressed cell viability and proliferation, and promoted apoptosis in human small cell lung cancer cells (H69AR) by regulation of miR-126/Akt/mTOR/PI3K pathway via miR-126 overexpression and suppression of VCAM-1, p-Akt, PI3K, NF-κB, and p-mTOR pathways (Chen et al., 2018). In one study, the antimetastatic properties of naringin were evaluated, and results showed that treating A549 lung cancer cells with naringin resulted in a reduction of cell invasion, cellular motility, cell viability, and cell-matrix adhesion (Hsiao et al., 2007b). In a separate study, treatment with naringin increased the inhibitory activity of trypsin and enhanced its cytotoxic and anti-platelet aggregation activity against A549 cells (Kim et al., 1998).

Ruthenium is a great alternative to platinum due to its extensive variety of oxidation states and its capability to form complexes with bioactive ligands (Jayakumar et al., 2018). Naringin was used to fabricate a ruthenium complex with anticancer activity. Results showed that a naringin-ruthenium (II) complex reduced cell viability and proliferation, and promoted apoptosis in A549 human lung adenocarcinoma (Garcia et al., 2019). Also, treatment with naringin alone and in combination with transition metal ions [Ag (I), Y (III), and Ru (III)] reduced cell growth and viability, and enhanced apoptosis in A549 human lung adenocarcinoma. Additionally, results showed that transition metal ions enhance the naringin activity when they are coordinated with each other (Atta et al., 2019). Another study showed that treatment of A549 cells with naringin promoted accumulation of GM3 through inhibition of NEU3 and resulted in the attenuation of ERK/EGFR signaling accompanied by a reduction in cell growth (Yoshinaga et al., 2016).

### Neuroblastoma

Neuroblastoma is the most frequent extracranial solid tumor in children. There is an increasing interest in using plant-derived dietary compounds for the treatment of several solid tumors, including malignant neuroblastoma (Yamane and Kato, 2012). Naringin treatment reduced cell viability and promoted apoptosis in rotenone-treated SH-SY5Y human neuroblastoma cell line through suppression of P38 and JNK phosphorylation, as well as activation of caspase-3 and caspase-9 (Kim et al., 2009).

### Ovarian Cancer

Ovarian cancer is a heterogeneous group of neoplasms, which is classified based upon type and degree of differentiation. It is one of the most deadly female reproductive system malignant tumors (Cho and Shih, 2009). The most efficient treatment for ovarian cancer is platinum-based chemotherapy and surgical cytoreduction (Jessmon et al., 2017). Resistance to platinum-based agents is one of the difficulties of ovarian cancer treatment using pharmacological agents (Choi et al., 2008). Therefore, investigating novel agents with low toxicity and high efficacy that can also reduce resistance to platinum-based agents, is very important. NF-κB is highly expressed in cisplatin-resistant ovarian cancer cell lines and has a crucial role in the drug resistance of ovarian cancer cells (Choi et al., 2008). One study demonstrated that the inhibitory effect of naringin was associated with inhibition of the NF-κB signaling pathway, and treatment with naringin significantly reduced P-glycoprotein (P-gp) and NF-κB expression in a concentration-dependent way in SKOV3/CDDP cisplatin-resistant human epithelial ovarian cancer cell line (Zhu et al., 2018). Another study also indicated that naringin downregulated COX-2 and NF-κB expression in a concentration-dependent way in SKOV3/CDDP cells (Zhu et al., 2017). In an *in vivo* study, treatment with naringin reduced ovarian tumor size and weight in tumor-bearing mice. Naringin also induced apoptosis by decreasing c-Myc, Bcl-2, surviving, cyclin D1, and Bcl-xL and increasing caspase-3 and caspase-7 levels in ovarian tumor cells. Such suppression may be related to the NF-κB signaling pathway regulation (Cai et al., 2018). Interestingly, at least one study indicated that naringin had no obvious inhibitory impact on cell growth and proliferation of OVCAR-3 human ovarian cancer cell line (Luo et al., 2008).

### Prostate Cancer

Prostate cancer is the second most common cancer in men and the fourth most common cancer overall (Ferlay et al., 2015). The combined consumption of nutraceutical agents and chemotherapeutic drugs is a great approach for increasing the therapeutic anticancer impacts, as well as easing adverse effects of chemotherapy and drug resistance (de Oliveira Júnior et al., 2018). Atorvastatin, a 3-hydroxyl-3-methylglutaryl coenzyme A reductase inhibitor, has demonstrated antitumor activity in prostate cancer (Allott et al., 2017). One study examined the anticancer effect of naringin in combination with atorvastatin on PC-3 and LNCaP prostate cancer cell lines. Results demonstrated that combined treatment of atorvastatin and naringin synergistically induced apoptosis, reduced cell growth, suppressed invasion and migration, and potently inhibited AR, p-STAT3, survivin, p-Akt, and Bcl-2 expression levels. Additionally, treatment with naringin alone or combined with atorvastatin suppressed the tumor growth in tumor-bearing SCID mice, and the combined treatment demonstrated a greater inhibitory effect compared to either compound alone (Wu et al., 2019). In another study, PC3, DU145, and LNCaP cell lines were treated with different concentrations of naringin, paclitaxel, and their combinations. Treatment with naringin individually or combined with paclitaxel suppressed cell proliferation and cell survival in a concentration- and time-dependent way through induction of apoptosis and cycle arrest as well as decreased cell migration via inhibition of NF-κB, ERK, and Akt signaling and upregulation of phosphatase and tensin homologue (PTEN) expression. Taken together, naringin synergistically promoted the paclitaxel cytotoxic impact in PCa cell lines (Erdogan et al., 2018). In an *in vitro* study, treatment with naringin decreased cell viability and proliferation as well as enhanced apoptosis in DU145 prostate cancer cell line. Naringin also enhanced oxidative stress and had a genotoxic effect on prostate cancer cells (Lewinska et al., 2015).

### Sarcoma

Even though adjuvant chemotherapy has led to the improved survival rates in osteosarcoma patients, the development of multidrug resistance has seriously influenced prognosis and limited the success of therapeutic attempts (Davis et al., 2018). Hence, novel and effective drugs for osteosarcoma treatment are required. One study showed that naringin treatment suppressed cell migration, invasion, and proliferation, and promoted apoptosis and cell cycle arrest in MG63 and U2OS human osteosarcoma cells through blockage of zinc finger E-box binding homeobox 1 (Zeb1), which plays a role in tumor metastasis. Additionally, naringin decreased tumor nodule formation and expression of MMP-2, Bcl-2, and cyclin D1 in the livers of mice bearing MG63 osteosarcoma cell line (Ming et al., 2018). However, another study indicated that naringin did not affect the MG-63 osteosarcoma cells’ growth rate (Zhang et al., 2018a). *In vitro* treatment of JJ012 human chondrosarcoma cells with naringin reduced cell invasion and migration through the suppression of VCAM-1 expression by enhancing miR-126 expression (Tan et al., 2014).

In an *in vivo* study, naringin treatment demonstrated significant inhibition of tumor growth in male ddY mice bearing S180 sarcoma cancer cells (Kanno et al., 2005). *In vivo* treatment of naringin decreased TNF-α and IL-6 levels, suppressed tumor growth, and increased the survival rate in Wistar rats bearing W256 carcinosarcoma cells (Camargo et al., 2012).

### Skin Cancer

Melanoma is the leading cause of mortality from skin cancer (Siegel and Naishadham, 2013). Historically, melanoma has been refractive to chemotherapy which provides very low response rates and very few beneficial effects in overall survival. Therefore, multiple targeted therapeutic approaches have been examined (Ascierto et al., 2013). Treatment of A375 and A875 melanoma cell lines with naringin promoted cycle arrest and apoptosis, and also suppressed cell proliferation and growth in a concentration-dependent way. Naringin also suppressed c-Src and cancer cell metabolism through suppression of the c-Src/Akt signaling pathway, leading to a decrease in cell migration and invasion (Guo et al., 2016). Another study demonstrated that naringin treatment reduced the metastatic foci formation and increased the survival rate in mice bearing B16FlO melanoma cells (Menon et al., 1995). Another study also indicated that *in vitro* treatment of naringin reduced cell invasion in MO4 mouse melanoma cell line (Bracke et al., 1991). In an *in vitro* study, the anticancer effect of the naringin-derived copper (II) complex was investigated. The results showed that treatment of B16FlO melanoma with naringin or naringin-Cu (II) complex reduced cell proliferation and growth, increased cell death, and decreased diphenylpicrylhydrazyl radical (DPPH). Additionally, it demonstrated that the naringin-Cu (II) complex had higher anti-inflammatory, antioxidant, and anticancer activities in comparison to free naringin without decreasing cell viability (Pereira et al., 2007).

### Stomach Cancer

Gastric cancer is the fourth most detrimental cancer-related death in the world (Torre et al., 2015). A large number of cancer cases and mortality could be avoided with early detection, using the phytomedicine intervention as an alternative to radiotherapy and chemotherapy. One study investigated the mechanism behind naringin-mediated autophagic cell death in AGS gastric cancer cell line. Naringin treatment promoted lysosomal membrane permeabilization through suppression of Akt/mTOR/PI3K signaling cascade, resulting in lysosomal cell death protein cathepsin D-mediated ERK1/2-p38 MAPKs activation through Bcl-xL decrease, and Bad, BH3, and ROS increase in autophagy-mediated cell death in AGS cell line. Additionally, rapamycin pre-treatment with naringin indicated a significant reduction in mTOR phosphorylation and enhancement in LC3B activation in AGS cells compared with naringin treatment alone (Raha et al., 2020). In another study, naringin treatment suppressed viability and growth in the AGS (human gastric epithelial 108 adenocarcinoma) cells (Hsiao et al., 2007a). Naringin treatment of AGS cells induced autophagy-mediated growth suppression through suppression of PI3K/Akt/mTOR cascade, and potentially via activation of MAPKs (Raha et al., 2015). Furthermore, treatment with naringin increased the inhibitory activity of trypsin and increased the cytotoxic activity and anti-platelet aggregation activity against SNU-1 human stomach cancer cells (Kim et al., 1998).

### Thyroid Cancer

Thyroid cancer is the most frequent malignant tumor of the endocrine system (Kweon et al., 2014). The current approach for thyroid cancer treatment includes thyroid hormone inhibition therapy, surgical treatment, adjuvant radiotherapy, and isotope iodine-131 therapy. However, there are several disadvantages for these different types of treatment (Tang et al., 2017; Zivancevic-Simonovic et al., 2017). Hence, the development of low-toxic, effective, and new inhibitors is important in improving the survival rates of thyroid cancer patients. One study showed that *in vitro* treatment of SW1736 and TPC-1 thyroid cancer cells with naringin reduced cell proliferation and promoted apoptosis via suppression of PI3K/Akt pathway (Zhou et al., 2019).

## Nanostructered Formulations of Naringin in Combating Malignancies

In the past two decades, nanotechnology-based delivery systems have gained interest as a method to overcome the challenges associated with solubility, bioavailability, distribution, low therapeutic index, toxicity and targeting of conventional chemotherapeutic drugs as well as anticancer natural compounds (Feng and Mumper, 2013; Davatgaran-Taghipour et al., 2017; Kashyap et al., 2021; Lagoa et al., 2020). Naringin is one of the most fascinating phytopharmaceuticals that has been broadly examined for different biological activities. Yet, its suboptimal bioavailability, limited permeability, and low water solubility have restricted its use. A useful approach to overcome these difficulties is encapsulation of the agent into different nano-sized delivery vehicles (Mohamed et al., 2018).

Gold-naringin nanoclusters (GNNC) showed cytotoxic effects against A549 lung cancer cells and reduced the cell viability with increased concentrations of GNNC. At the same time, the WI-38 levels in lung normal cells remained elevated, even after treatment with high doses of GNNC (Sangubotla et al., 2020) (Table 5).

**TABLE 5:** Anticancer effects of naringin-based nano-drug delivery systems.

Nano-formulation	Cancer type	Study type	Cell line/animal model	Dose/Conc	Source	Purity (%)	Quality control reported? (Y/N)	Duration	Outcomes	References
Gold-naringin nanoclusters	Lung	*In vitro*	A549 cell line	15–90 μg/ml	Sigma-Aldrich (St. Louis, MO, United States)	ND	Y	1–5 days	↓Cell viability	Sangubotla et al. (2020)
Naringin-reduced graphene oxide nanosheets	Colon	*In vitro*	HT-29 cell line	0.39–12.5 μM	KPI. (Shanghai, China)	ND	Y	24 h	↓Cell growth, ↓cell proliferation, ↑apoptosis	Han et al. (2020)
Ti-Naringin-PBA-ZnO nanoparticles	Osteosarcoma	*In vitro*	Saos-2 cell line	98.6 μg/ml	Aladdin industrial co. Ltd. (Shanghai, China)	ND	Y	1–7 days	↑Apoptosis, ↑ROS, ↑MAPK/ERK pathway	Yang et al. (2020)
Nanostructured lipid carrier-containing naringin and coix seed oil	Liver	*In vitro; In vivo*	HepG2 cell line; BALB-nu nude mice with HepG2 xenografts	0.39–25 μM; 20 mg/kg	Shanghai Standard Technology Co. Ltd. (Shanghai, China)	(>98%)	Y	1–10 days	↓Cell proliferation, ↑apoptosis, ↓cell viability, ↓tumor growth	Zhu et al. (2020)
PTX-NRG-MIC micelles	Breast	*In vitro*	MCF-7 cell line	15–100 mg/ml	Sigma-Aldrich (Taufkirchen, Germany)	ND	Y	4–24 h	↓Cell growth, ↓cell viability, ↑intracellular uptake	Jabri et al. (2019)
Naringin-PF68 micelles	Liver, breast and colorectal	*In vitro; In vivo*	Caco-2, HepG2, and MCF-7 cell lines; female Swiss albino mice with EAC cells	0.1–40 μM; 100 mg/kg	Sigma-Aldrich co. (St louis, MO, United States)	ND	Y-HPLC	1–7 days	↓Tumor growth, ↓cell viability, ↓cell proliferation, ↓Tumor size	Mohamed et al. (2018)
PLGA nanoparticles co-encapsulating naringin and celecoxib	Lung	*In vitro*	A549 cell line	0.78–100 μM	Sigma-Aldrich (St. Louis, MO, USA)	ND	Y	10–70 h	↓Cell viability, ↓cell proliferation, ↑apoptosis	Said-Elbahr et al. (2016)
Naringin-conjugated gold nanoparticles	Breast and prostate	*In vitro*	MCF-7, MDA-MB-231, T47D, and PC-3 cell lines	30–100 μg/ml	Sigma-Aldrich (Chandigarh, India)	ND	Y	24 h	↓Cell viability, ↓cell proliferation	Singh et al. (2016)

**Note:** A down arrow indicates a reduction or decrease and an up arrow indicates an increase. EAC, Ehrlich ascites carcinoma; ERK, extracellular signal-regulated kinase pathway; MAPK, mitogen-activated protein kinase; ND, not determined; PLGA, poly D,L-lactide-co-glycolic acid copolymer; PTX-NRG-MIC, paclitaxel- and naringin-loaded mixed micelles; ROS, reactive oxygen species; Ti-naringin-PBA-ZnO, titanium-naringin-3-carboxyphenylboronic acid-zinc oxide.

With advances in nanotechnology and the extensive use of graphene, it has become essential to evaluate the possible disadvantages of graphene. Thus, most of the current studies are focused on different modified graphene. Naringin-reduced graphene oxide nanosheets (rGO@Nar), promotes cytotoxicity in the colon cancer cells (HT-29) through increased apoptosis and reduced cell viability and proliferation. The rGO@Nar plus naringin is more efficient toward colon cancer in comparison to rGO@Nar or naringin alone. Additionally, it has been shown that rGO@Nar together with naringin and rGO@Nar can efficiently eliminate tumor cells without affecting normal cells (Han et al., 2020). rGO@Nar may be a promising agent for assessment in the *in vivo* models of colon cancers.

After bone tumor resection, the large deficits are normally reconstructed with titanium (Ti)-based metallic endoprosthesis. When applied in osteosarcoma resection, Ti implant-related infection and tumor recurrence were determined as two crucial factors for failure of implantation. Ti-naringin-3-carboxyphenylboronic acid (PBA)-zinc oxide (ZnO) nanoparticles reduce infection and tumor recurrence and induce Saos-2 osteosarcoma cells apoptosis through activation of ROS/ERK signaling. *In vitro* cellular experiments showed that these nanoparticles could promote the proliferation and growth of osteoblasts (Yang et al., 2020). Nevertheless, *in vivo* experiments are required for understanding the anticancer properties of Ti-Naringin-PBA-ZnO nanoparticles in osteosarcoma.

Lipid-based nanoparticles are another delivery system that has particular benefits due to their unique properties, such as biodegradation, biological compatibility, multiple routes of administration, and convenient and easy industrial scale-up process (Rout et al., 2018). Nanostructured lipid carriers containing naringin and coix seed oil (NCNLCs) were successfully fabricated, and their cytotoxic activity was evaluated. NCNLCs reduced proliferation and viability and promoted apoptosis in HepG2 liver cancer cells and had a greater cytotoxic effect compared with free naringin, NONLCs, and NDNLCs. Moreover, the *in vivo* synergistic anticancer efficacy was evaluated in NCNLCs in xenograft tumor mice models and results showed that NCNLCs upregulated the IL-10 and IL-6 expression in the serum of tumor-bearing mice and inhibited tumor growth (Zhu et al., 2020).

The development of multidrug resistance (MDR) has restricted the efficacy of chemotherapeutic agents. Co-delivery of natural flavonoids with anticancer drugs in polymeric micelles is a potentially significant approach for overwhelming MDR and enhancing their anticancer efficacy. Paclitaxel co-encapsulation with naringin in mixed polymeric micelles increased *in vitro* cytotoxicity toward MCF-7 breast cancer cell line. Paclitaxel- and naringin-loaded mixed (PTX-NRG-MIC) micelles synergistically reduced the growth and viability of MCF-7 cell line and increased their intracellular uptake. Additionally, PTX-NRG-MIC micelles are more effective toward breast cancer when compared to naringin or paclitaxel (Jabri et al., 2019). Yet, *in vivo* experiments are required to confirm the active targeted delivery of PTX-NRG-MIC micelles.

Naringin polymeric micelles based on pluronic F68 were fabricated and their *in vitro* cytotoxicity was assessed against different cancer cell lines. Naringin-PF68 micelles reduced the viability and proliferation of Caco-2, HepG2, and MCF-7 cells. Furthermore, Swiss albino mice were used to evaluate the anticancer activity of naringin-PF68 micelles compared to the free drug. Results showed that PF68 micelles of naringin reduced tumor size and inhibited tumor growth in tumor-bearing mice. Naringin-PF68 micelles had a greater cytotoxic effect when compared with free naringin (Mohamed et al., 2018).

In another study, poly D,L-lactide-co-glycolic acid copolymer (PLGA) nanoparticles co-encapsulating celecoxib and naringin were synthesized and induced apoptosis and inhibited proliferation in the A549 cells in a concentration-dependent manner. They also showed greater cytotoxic activity on A549 cells in comparison to the combination of free drugs, while exhibiting significant safety on healthy lung tissues (Said-Elbahr et al., 2016).

In another study, gold nanoparticles (AuNPs) were synthesized using naringin as a reducing and stabilizing agent to create nano-theranostic agents. Naringin-conjugated gold nanoparticles (N-AuNPs) were evaluated with cytotoxicity and hemolysis assays, which showed their biocompatibility with MDA-MB-231 cell lines and normal red blood cells, while also demonstrating their potential to induce cell death in T47D, PC-3, and MCF-7 cell lines. *In vivo* studies must be conducted to confirm the active targeted delivery of N-AuNPs in cancer (Singh et al., 2016).

Taken together, results show that the nano-drug delivery systems have the capability to overwhelm the pharmacokinetic restrictions of naringin, highlighting its impacts on cancer therapy. Further research is required for designing surface-modified nano-formulations of naringin to achieve adjusted drug delivery systems.

## Pharmacokinetics and Toxicity of Naringin

After a single oral administration (42 mg/kg) of naringin in aged rats, minor differences were shown in the area under the plasma concentration-time curve (AUC) of total naringenin and naringin gained in the gastrointestinal tract, stomach, liver, muscle, kidney, and brain between male and female aged rats. It must be mentioned that the AUC of naringin in the trachea (3,140%) and lung (1,250%) of female rats in comparison to male rats were considerably higher, indicating that naringin may exhibit gender differences in the treatment of respiratory diseases in elderly individuals (Zeng et al., 2019). In multiple-dose studies, no considerable accumulation of naringenin was detected in rats, dogs, and humans. In single-dose studies, various pharmacokinetic parameters in females, including the elimination half-life (t_1/2_) (naringenin, rats, oral, 42 mg/kg), AUC (naringin, humans, 160 mg (high-fat diet); naringin, rats, oral, 21 mg/kg; and naringenin, humans, 40 mg), peak plasma concentration (C_max_) (naringenin, humans, 40 mg), and time to reach *C*_max_ (T_max_) (naringenin, rats, oral, 168 mg/kg) were considerably higher than those of males, while a small number of pharmacokinetic parameters in females, including AUC (naringin, humans, 40 mg) and C_max_ (naringenin, rats, i.v., 42 mg/kg) were significantly lower than those of males. In multiple-dose studies, considerably greater female parameters were only detected in rats (naringenin, accumulation index, 1.79 ± 0.457 and naringin, T_max_, 2.70 ± 1.48 h) (Bai et al., 2020). The plasma drug concentration-time curves indicate that the oridonin AUC 0–24 h value was nearly three times larger compared to naringin, while the naringin dose was approximately four times larger compared to oridonin, indicating that the oridonin absorption in rats is higher than naringin (Jin et al., 2015). Naringin administration (15 mg/kg) suppressed the P-gp function and considerably enhanced the candesartan intestinal absorption by 3.2 times (Surampalli et al., 2015). After administration of 600 and 1,000 mg/kg naringin through the duodenal cannula, the naringin average C_max_ in portal plasma was measured at 18.8 ± 3.8 min, while the naringin absorption ratios in lymph fluid and portal plasma were nearly 4.1 and 95.9, respectively. This suggests that naringin may be absorbed through portal blood and concentrations may be reduced via bile excretion (Tsai and Tsai, 2012). In membrane toxicity studies, naringin administration (15 mg/kg, w/w) did not cause any toxicity; but, it insignificantly increased the protein release from the intestinal membrane (Surampalli et al., 2015). The sub-chronic and acute toxicology of naringin was indicated to be almost non-toxic for Sprague-Dawley rats, and the naringin no-observed-adverse-effect-level (NOAEL) in rats was larger than 1,250 mg per kg per day after oral administration for 13 weeks (Li et al., 2013b). No intestinal membrane impairment was noted in the naringin presence through measurement of the alkaline phosphatase and protein release (Surampalli et al., 2015).

## Conclusions and Future Directions

**FIGURE 3:**
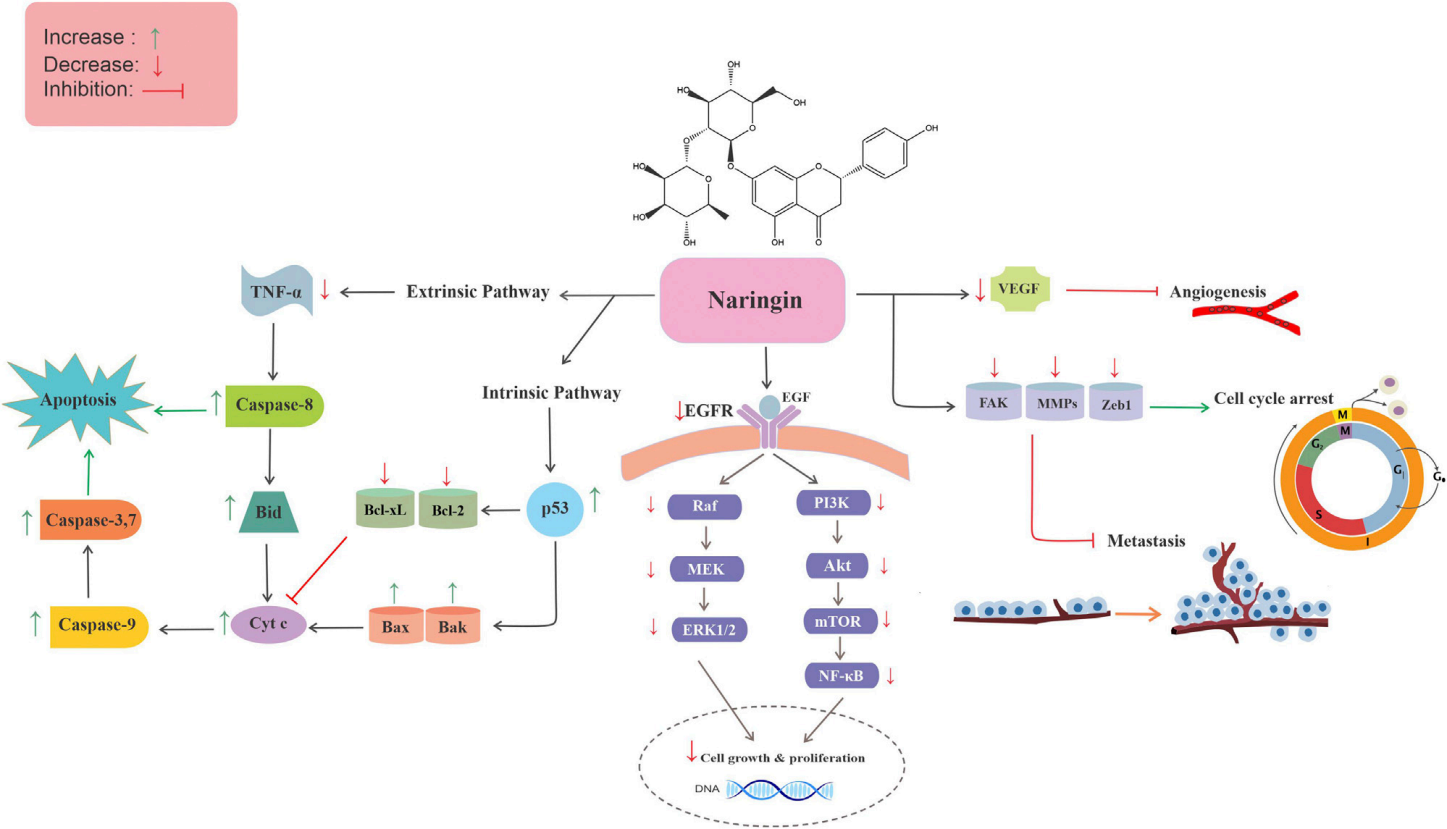
Molecular mechanisms underlying anticancer effect of naringin. Bax, Bcl-2-associated X protein; Bak, Bcl-2 homologous antagonist/killer; Bcl-2, B-cell lymphoma 2; Bcl-xL, B-cell lymphoma-extra-large; Bid, BH3-interacting domain death agonist; cyt. c, cytochrome c; EGF, epidermal growth factor; EGFR, epidermal growth factor receptor; ERK1/2, extracellular signal-regulated kinase 1 and 2; FAK, focal adhesion kinase; MEK, mitogen-activated protein kinase; MMPs, matrix metallopeptidases; mTOR, mammalian target of rapamycin; NF-κB, nuclear factor-κB; p53, tumor protein p53; PI3K, phosphatidylinositol-3-kinase; Raf, rapidly accelerated fibrosarcoma; TNF-α, tumor necrosis factor-α; VEGF, vascular endothelial growth factor; Zeb1, zinc finger E-box binding homeobox 1.

Natural products have played an important part in the treatment of human diseases and most notably, in cancer therapies. Naringin, a flavone glycoside, is promising for the treatment of many diseases due to its low cost, broad availability, long history of use, and variety of pharmacological actions, with the predominant evidence currently focusing on its anticancer impacts. Naringin alone, or combined with other drugs may be useful for treating cancers. Emerging studies showed that naringin-metal complexes have greater anticancer activities compared to free naringin. Naringin can impact several cancer types, including glioblastoma, hepatocellular carcinoma, lung adenocarcinoma, breast cancer, prostate cancer, melanoma, leukemia, colon cancer, gastric cancer, oral cancer, brain cancer, bladder cancer, and ovarian cancer. It has been demonstrated that naringin employs multiple mechanisms to hamper cancer initiation, promotion, and progression via modulation of several dysregulated signaling cascades implicated in inflammation, proliferation, cell survival, apoptosis, autophagy, angiogenesis, invasion, and metastasis (Figure 3). In particular, the cancer-inhibitory effects of naringin have been linked to the regulation of various signaling pathways, such as Nrf2, NF-κB, PI3K/Akt/mTOR, JNK, ERK, and p38 MAPK. Naringin intervenes with the function of various signaling molecules, such as caspases, Bax, TNF-α, Bcl-2, VEGF, and ILs. Various patents have shown that naringin can specifically affect desired targets (Table 6), making it possible to use naringin and its formulations for further investigations for targeted therapies.

**TABLE 6:** Anticancer effects of naringin based on google patents and US patents registry.

Patent NO	Cancer types	Subjects	Results	Major outcomes	References
ES2519040T3	Liver and lung cancers	Cell lines, animals, and humans	Inhibiting TGF-β1 signaling pathway; improving serum IFN-γ	Treating or preventing fibrosis and tumors	Liang et al. (2008)
US7326734B2	Bladder cancer	Cell lines	Inhibiting cell proliferation	Treating or preventing tumors	Zi et al. (2004)
US10307393B2	Pulmonary carcinoma, esophageal carcinoma, breast carcinoma, and mediastinum tumors	Cell lines and animals	Reducing the release of the inflammatory factors (IL-1β, IL-6, TNF-α, TGF-β, and IFN-γ)	Radiotherapy protection and treating tumors	Liang et al. (2016)
JP2005508312A	Colorectal, cervical, gastric, lung cancer, malignant glioma, ovarian, and pancreatic cancers	Cell lines, animals, and humans	Inhibiting MDR1 gene expression	Treating or preventing tumors	Gunther and Reinhold, (2005)
KR20060120101A	Prostate, colorectal, and liver cancers	Cell lines	Binding to EGR-1-like promoter sequences to modulate the expression of cancer related genes (p21 and p53)	Treating or preventing tumors	Wong et al. (2004)

EGR1, early growth response 1; IFN-γ, interferon-γ; IL-1β, interleukin-1β; IL-6; interleukin-6; MDR1, multi-drug resistance-1; TGF-β1, transforming growth factor-β1; TNF-α, tumor necrosis factor-α.

The biggest obstacle concerning naringin as a novel therapeutic agent is that current naringin anticancer evidence is focused on *in vitro* models of cancers, and there are few detailed *in vivo* studies. Another challenge is related to bioavailability of naringin and selection of a dose range for clinical/therapeutic application based on available preclinical studies. Nevertheless, according to completed and undergoing clinical studies on naringin, bioavailability and effective concentration may not be barrier for exploring its therapeutic benefits. Moreover, since all studies presented in this review utilized pure compounds obtained from various sources, providing a full taxonomic validation of the material under investigation is one of the challenges. The possible restrictions with naringin pharmacokinetics highlight the necessity for the development of novel delivery systems. In particular, further experimental and technological approaches are needed for designing surface-modified naringin-nanostructures to attain targeted drug delivery systems toward cancer. The result of research findings as evaluated here might offer substantial support for further development of naringin as a multi-targeted agent for the prevention and intervention of human malignancies.

## Data Availability

The original contributions presented in the study are included in the article/Supplementary Material, further inquiries can be directed to the corresponding authors.
